# Phosphoprotein enriched in diabetes (PED/PEA15) promotes migration in hepatocellular carcinoma and confers resistance to sorafenib

**DOI:** 10.1038/cddis.2017.512

**Published:** 2017-10-26

**Authors:** Cristina Quintavalle, Sravanth Kumar Hindupur, Luca Quagliata, Pierlorenzo Pallante, Cecilia Nigro, Gerolama Condorelli, Jesper Bøje Andersen, Katrin Elisabeth Tagscherer, Wilfried Roth, Francesco Beguinot, Markus Hermann Heim, Charlotte Kiu Yan Ng, Salvatore Piscuoglio, Matthias Sebastian Matter

**Affiliations:** 1 Institute of Pathology, University Hospital of Basel, Basel, Switzerland; 2 Biozentrum, University of Basel, Basel, Switzerland; 3 Istituto per l’Endocrinologia e l’Oncologia Sperimentale (IEOS), ‘G. Salvatore’, Consiglio Nazionale delle Ricerche (CNR), Naples, Italy; 4 URT of the Institute of Experimental Endocrinology and Oncology ‘G. Salvatore’, National Council of Research, Naples, Italy; 5 Department of Translational Medical Sciences, University of Naples ‘Federico II’, Naples, Italy; 6 Dipartimento di Medicina Molecolare e Biotecnologie Mediche (DMMBM), Università degli Studi di Napoli ‘Federico II’, Naples, Italy; 7 Biotech Research and Innovation Centre, University of Copenhagen, Copenhagen, Denmark; 8 Institute of Pathology, University Medical Center Mainz, Mainz, Germany; 9 Division of Gastroenterology, University Hospital of Basel, Basel, Switzerland

## Abstract

Hepatocellular carcinoma (HCC) is the third-leading cause of cancer-related death with limited treatment options and frequent resistance to sorafenib, the only drug currently approved for first-line therapy. Therefore, better understanding of HCC tumor biology and its resistance to treatment is urgently needed. Here, we analyzed the role of phosphoprotein enriched in diabetes (PED) in HCC. PED has been shown to regulate cell proliferation, apoptosis and migration in several types of cancer. However, its function in HCC has not been addressed yet. Our study revealed that both transcript and protein levels of PED were significantly high in HCC compared with non-tumoral tissue. Clinico-pathological correlation revealed that PED^high^ HCCs showed an enrichment of gene signatures associated with metastasis and poor prognosis. Further, we observed that PED overexpression elevated the migration potential and PED silencing the decreased migration potential in liver cancer cell lines without effecting cell proliferation. Interestingly, we found that PED expression was regulated by a hepatocyte specific nuclear factor, HNF4*α*. A reduction of HNF4*α* induced an increase in PED expression and consequently, promoted cell migration *in vitro*. Finally, PED reduced the antitumoral effect of sorafenib by inhibiting caspase-3/7 activity. In conclusion, our data suggest that PED has a prominent role in HCC biology. It acts particularly on promoting cell migration and confers resistance to sorafenib treatment. PED may be a novel target for HCC therapy and serve as a predictive marker for treatment response against sorafenib.

Hepatocellular carcinoma (HCC) is the third most common cause of cancer-related mortality worldwide.^1, 2^ Unlike most other malignancies, mortality from liver cancer has increased significantly over the past 20 years and the medical and economic burden of liver cancer will likely increase significantly in Western populations over the next decades.^3^ Prognosis of HCC patients is poor and 5-year survival rate is around 15%.^2^ Risk factors for HCC development include chronic viral infection with Hepatitis B or C virus (HBV/HCV), excessive alcohol abuse and non-alcoholic steatohepatitis due to obesity and diabetes.^2^ Patients are only eligible for potentially curative treatments such as surgical resection or liver transplantation if HCC is detected at an early stage.^2^ For systemic treatment, the only drug currently approved is sorafenib, a multi-tyrosine kinase inhibitor.^2^ Although sorafenib has been shown to increase overall survival in patients with advanced stage HCC, response rate is poor,^4^ with only 2% of the patients achieving partial response, 71% achieving stable disease and no patients achieving complete response in the phase 3 SHARP trial (*n*=602).^4^ Resistance to sorafenib in HCC and other cancers have been associated, for example, with elevated expression of angiogenesis-related genes, fibroblast growth factor-1, NF-*κ*B, upregulation of the targeted MAPK/ERK pathway and epithelial–mesenchymal transition.^5, 6, 7, 8^ In addition, sorafenib is frequently accompanied by moderate to severe side effects.^2^ Therefore, it will be beneficial to identify patients who may benefit from sorafenib therapy and to improve our understand of the mechanisms of sorafenib resistance.

PED (phosphoprotein enriched in diabetes), also known as PEA15 (phosphoprotein enriched in astrocytes 15), is a ubiquitously expressed phosphoprotein, which was originally identified in primary cultured astrocytes.^9, 10^ PED has a prominent role in diabetes and glucose metabolism.^11^ Furthermore, it modulates cellular processes such as proliferation, apoptosis and migration in various cancer types (e.g., breast, colon and esophageal cancers).^11, 12^ Interestingly, PED may act as a tumor promotor or tumor suppressor and this function seems to depend on its phosphorylation status.^13^ In its unphosphorylated form, PED binds ERK1/2 protein and prevents its subsequent activation. By contrast, phosphorylation of PED at Ser104 and Ser116 releases ERK1/2, which in turn leads to tumor promotion with increased cell proliferation and migration.^12^ In addition, PED phosphorylation at Ser116 facilitates its binding to Fas-associated death domain protein (FADD). Consequently, FADD-mediated apoptosis is prevented and results in cell growth advantage.^11^ PED levels are regulated by ubiquitination and proteasomal degradation.^14^ Furthermore, transcription factor HNF4*α* has been described as an upstream regulator of PED. By binding to the PED promoter, HNF4*α* suppresses PED expression.^15, 16^

Although the function of PED has been described in several tumor entities, its role in HCC is currently unknown. Therefore, we sought to determine PED expression in human HCC tissue samples and analyze its functional role by performing *in vitro* experiments. Additionally, we investigated its regulation, and the impact of PED expression on sorafenib therapy.

## Results

### PED expression is increased in HCC

**Figure 1:**
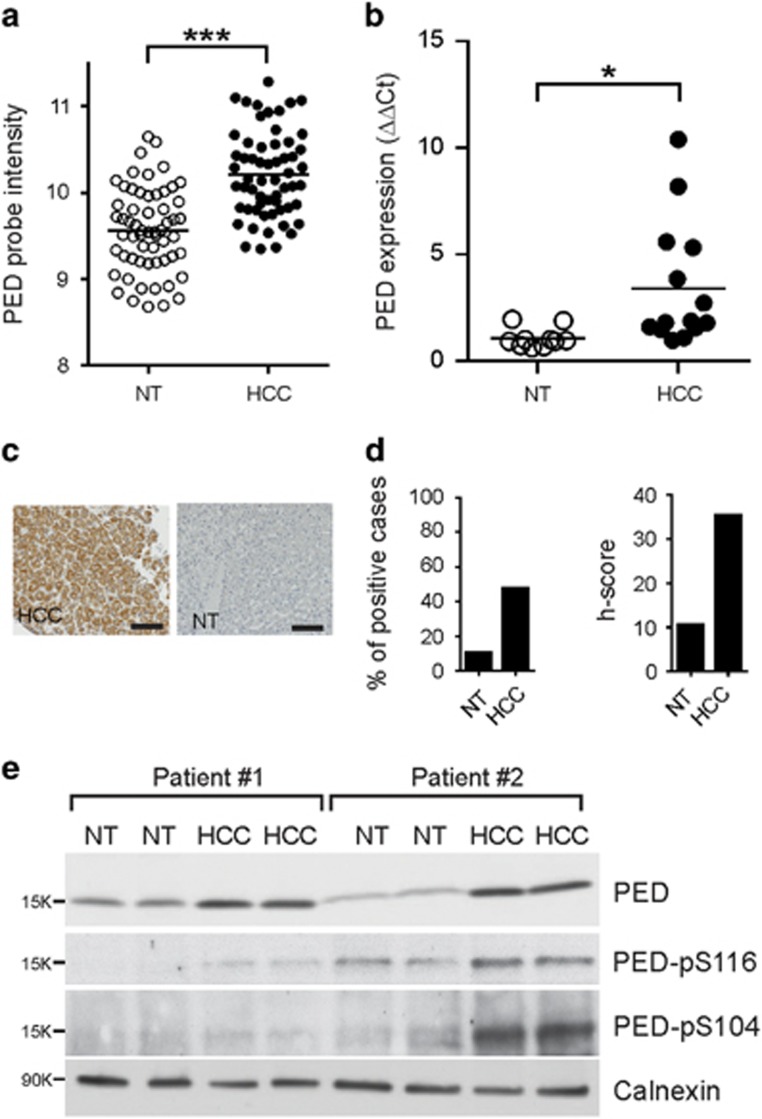
PED is overexpressed in HCC samples. (**a**) PED expression levels in HCC samples and their matched non-tumoral (NT) counterpart measured by an mRNA gene expression microarray. Data are reported as probe intensity. (**b**) PED mRNA was measured by qRT-PCR in a separate cohort of 14 HCC patients and compared with the 10 available non-tumoral counterpart. 18 S was used as internal control and 2^−ΔΔCt^ formula was applied to determine relative expression levels. Statistical analysis (**a**,**b**) with paired Student *t*-test. (**c**) Representative immunohistochemical staining from an HCC tumor (left) with positive (3+) PED staining and non-tumoral liver tissue (NT) showing negative PED staining (right). Scale bar=20 *μ*m. (**d**) Percentage and *h*-score (staining intensity × percentage of positive tumor cells) of PED positivity in HCC samples and non-tumoral (NT) liver tissues by immunohistochemistry. (**e**) Western blot analysis of total PED and phosphorylated PED (PED S116 and PED S104) in two HCC patient samples and their corresponding NT control tissues. Calnexin was used as internal control. *P<0.05, ***P<0.001

To determine the expression level of *PED* in HCC we re-analyzed a published gene expression microarray data set previously performed at our hospital containing human HCC samples and their corresponding non-tumoral liver tissues (*n*=59 pairs).^17^ The mean age of the HCC patients was 64 years. 88% of patients were male, had underlying liver cirrhosis and suffered from chronic viral liver disease (HCV and/or HBV) or alcohol abuse.^17^ Mean PED expression in the tumors was significantly elevated compared with the matched non-tumoral liver tissues (Figure 1a). However, not all HCC samples showed an increase of PED expression compared with the matched non-tumoral liver tissues, with 28.8% of the tumor samples showing an increase of two-fold or higher in comparison to the matched non-tumoral counterparts. To confirm the microarray results, we measured PED mRNA expression by qRT-PCR in the same cohort of patient samples with sufficient RNA left (*n*=24 paired). Consistently, PED mRNA expression in the tumors was significantly higher than the non-tumoral liver tissue (Supplementary Figure 2). In addition, we measured PED mRNA expression by qRT-PCR in HCC tumor samples of an independent patient cohort (*n*=14). The patients had a mean age of 69 years. 79% of the patients were male, had underlying liver cirrhosis and suffered from chronic viral liver disease (HCV and/or HBV) or alcohol abuse. In comparison to the non-tumoral liver tissues (*n*=10) and in line with the microarray results, PED expression was again increased in the HCC samples (Figure 1b) and 43% of the tumor samples showed an increase of two-fold or more in comparison to the mean of PED expression in the non-tumoral tissues. Furthermore, we performed immunohistochemistry for PED on a tissue microarray (TMA) containing formalin fixed and paraffin embedded HCC samples (*n*=45) and non-tumoral control liver tissue (*n*=20) (Table 1). Cytoplasmic staining intensity was graded as ‘0’ for negative staining, ‘1’ for weak, ‘2’ for moderate and ‘3’ for strong staining (Figure 1c; Supplementary Figure 1). PED was expressed (staining intensity 1, 2 or 3) in almost half (47%) of the HCC samples and less frequently in the non-tumoral liver tissues (15%) (Figures 1c and d). In addition, we determined the percentage of cells with positive staining to calculate the *h*-score (staining intensity × percentage of positive tumor cells). Consistently, the *h*-score was significantly higher in the HCC samples than in the non-tumoral control liver tissues (Figure 1d). In accordance, western blot analysis revealed a higher level of total PED in HCC (*n*=7) compared with the adjacent non-tumoral liver (Figures 1e and 4d; Supplementary Figure 2). Interestingly, PED was increased in its bi-phosphorylated form with phosphorylation at both Ser104 and Ser116 residues (Figure 1e).

In conclusion, our data demonstrate higher PED expression in HCC samples in comparison to non-tumoral liver tissue at mRNA and protein levels.

### PED is associated with metastasis formation and poor prognosis of HCC patients

**Figure 2:**
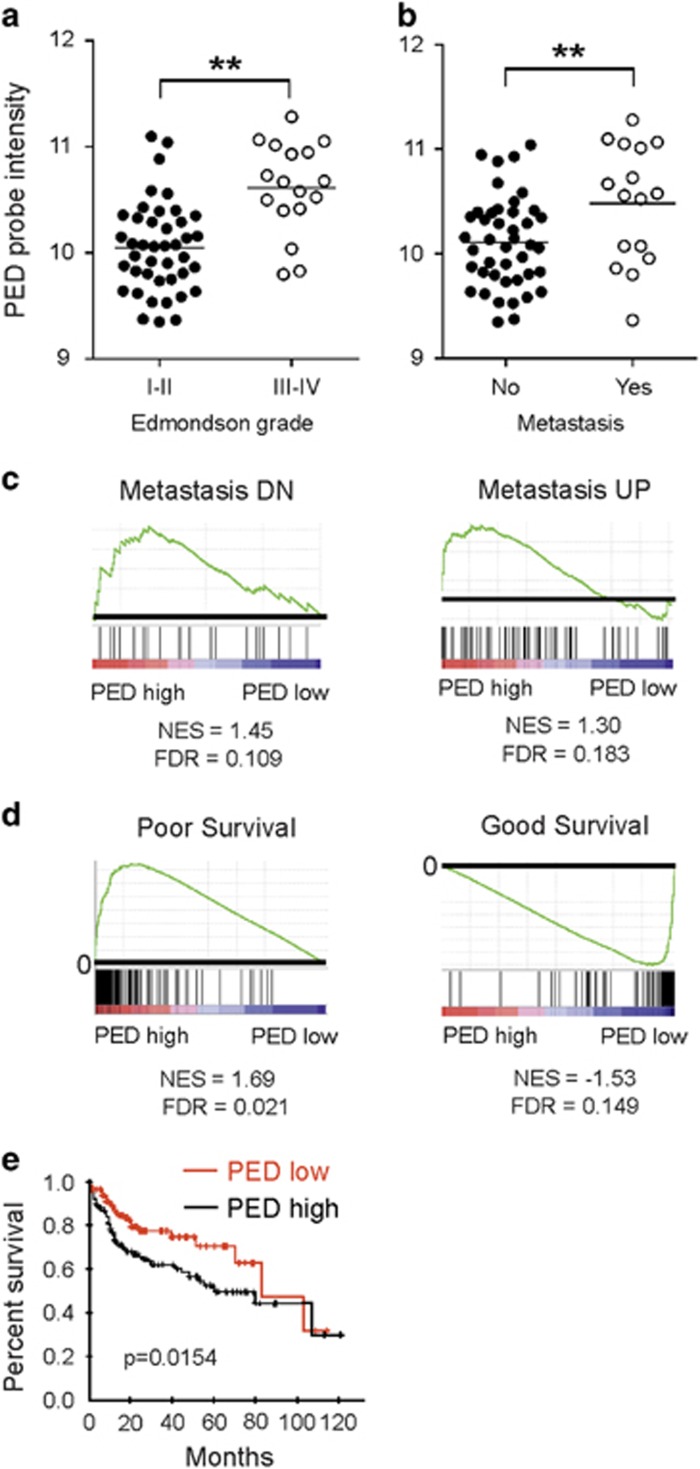
PED is associated with metastasis formation and poor patient survival. PED probe intensities from the gene expression microarrays of 59 HCC samples were compared between (**a**) those with low (I–II) or high (III–IV) Edmondson grades, and between (**b**) those with or without metastasis at the time of diagnosis. Statistical analysis (**a**,**b**) with Mann–Whitney *U*-test. (**c**) GSEA using a HCC metastasis-associated gene signature^18^ with downregulated (Metastasis DN) or upregulated (Metastasis UP) genes between HCC samples with high PED expression (PED high) or low PED expression (PED low). (**d**) GSEA using a gene signature from HCC patients with poor or good survival^19^ between HCC samples with high PED expression (PED high) or low PED expression (PED low). NES: normalized enrichment score. FDR: false discovery rate. (**e**) Survival analysis (Kaplan–Meyer) of HCC patients by calculating distribution in a previously published data set (Bioprofiling.de^20^) after stratification for high (*n*=127) and low (*n*=112) tumoral PED expression. ** *P*<0.01

Next, we correlated PED expression in the gene expression microarray data generated from the 59 patients with clinico-pathological data. PED was significantly (*P*<0.0001; Mann–Whitney *U*-test) overexpressed in poorly differentiated HCCs (Edmondson grades III and IV) than in well-differentiated HCCs (Edmondson grades I and II; Figure 2a). Interestingly, PED was also significantly overexpressed (*P*=0.014, Mann–Whitney *U*-test) in patients who had metastasis at the time of biopsy (Figure 2b). In accordance, gene set enrichment analysis (GSEA) using two previously published metastasis-associated gene signatures derived from HCC tumor samples^18^ showed significant enrichment in tumor samples with high PED expression (PED^high^, Figure 2c). In addition, a gene signature associated with poor survival in HCC patients^19^ was enriched in PED^high^ samples (Figure 2d). By contrast, a gene signature associated with good survival was enriched in samples with low PED expression (PED^low^). In line with these results, survival analysis using data from TCGA (Bioprofiling.de^20^) revealed a significant worse survival with PEDhigh (*n*=133) tumors in comparison to PED^low^ tumors (*n*=112) in a subgroup of patients (*n*=252) with N0 tumor stage (Figure 2e, *P*=0.0154). Association with worse survival was also observed in subgroups of patients characterizied by a T3 stage (PED^high^ *n*=23 *versus* PED^low^ *n*=20 *P*=0.0204), M0 stage (PED^high^ *n*=133 *versus* PED^low^ *n*=112 *P*=0.0196) and IIIa stage group (PED^high^ *n*=33 *versus* PED^low^ *n*=27 *P*=0.048). However, survival analysis covering all patients included by TCGA (*n*=442) and also with our cohort of 59 patients did not reveal a significant association of PED expression with patient survival (data not shown). Altogether, these results demonstrate that high *PED* expression is associated with high edmondson grade, metastasis formation and at at least in part with poor survival.

### PED promotes cell migration

**Figure 3:**
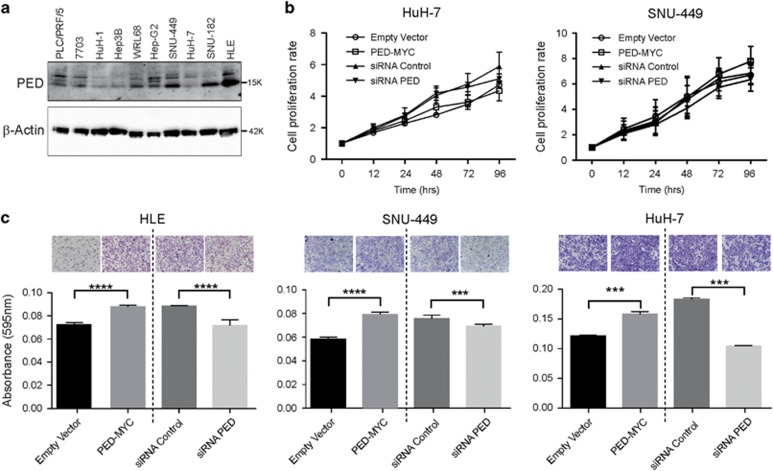
PED modulates cell migration. (**a**) Western blot analysis of PED protein expression in 10 different HCC cell lines. *β*-Actin was used as loading control. (**b**) HuH-7 and SNU-449 cells were transfected with PED-MYC or an empty control vector as wells as with siRNA against PED (siRNA PED) or control siRNA. Cell growth properties were evaluated by using xCELLigence instrument at the time indicated. Data are reported as mean±S.D. of two independent experiments performed at least in triplicate. Difference was evaluated between PED overexpressing (PED-MYC), PED silenced (siRNA PED), empty vector transfected and a siRNA control transfected cells (two-way ANOVA test). (**c**) HLE, SNU-449 and HuH-7 cell lines were transfected with a vector overexpressing PED (PED-MYC) or empty control vector, siRNA against PED (siRNA PED) or siRNA control. Migration was assessed using a transwell assay after 24 h. One representative image of crystal violet stained cells at 100 × is shown above and quantification by colorimetry below. ***P<0.001, ****P<0.0001

To gain insight into the functional role of PED in hepatocarcinogenesis, we performed *in vitro* experiments. First, we measured PED protein expression by western blot in ten different liver cancer cell lines (Figure 3a, quantification Supplementary Figure 3A). PED expression was variable among these cell lines and for example, SNU-449, SNU-182 and HLE cells showed high PED expression, whereas Hep3B and HuH-1 cells had low PED expression. In addition, we measured PED mRNA expression by qRT-PCR in 21 different liver cancer cell lines, which revealed similar variability of PED expression (Supplementary Figure 3B).

For functional analysis, we overexpressed PED by transfection with a vector (PED-MYC-tagged) and reduced PED expression by siRNA (Supplementary Figures 3C,D). We first measured cell proliferation, which remained unchanged after modulating PED expression in HuH-7 and SNU-449 cell lines (Figure 3b). By contrast, cell migration, as assessed by transwell plates, was promoted after overexpressing PED in HLE, SNU-449 and HuH-7 cell lines (Figure 3c) and cell migration was decreased after silencing PED by siRNA (Figure 3c).

Therefore, our data suggest that PED in HCC has a role in cell migration, which may contribute to metastasis formation. In contrast, no action recognized on cell growth.

### PED expression is regulated by HNF4*α*

**Figure 4:**
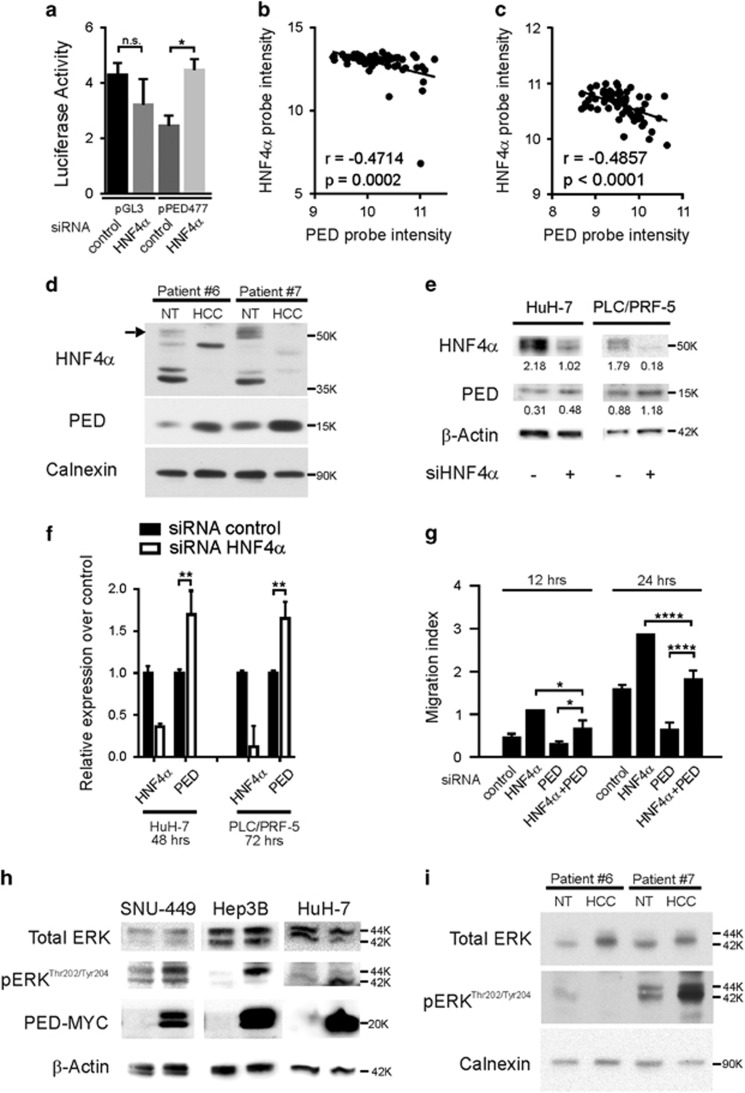
PED is inversely correlated to HNF4*α* expression. (**a**) SNU-449 cells were co-transfected with 100 ng of pPED477 PED promoter-luciferase or pGL3 basic construct and treated with siRNA against HNF4*α* or siRNA control. Luciferase activity was normalized for Renilla activity and is presented as mean±S.D. A representative experiment in triplicate is shown. (**b**,**c**) PED expression levels in HCC samples (**b**; *n*=59) or corresponding non-tumoral liver tissue (**c**, *n*=59) were correlated with HNF4*α* expression. Correlation was calculated by Spearman test. Data are reported as probe intensity of an mRNA transcriptome array. (**d**) Western blot analysis for HNF4*α* and PED in two HCC patient tumor samples and their corresponding non-tumoral (NT) tissues. Calnexin was used as loading control. Arrow: canonical full length HNF4*α* (52 kDa); other bands are isoforms or truncated forms of the protein. (**e**,**f**) HuH-7 and PLC/PRF/5 cell lines were transfected with siRNA against HNF4*α* (siHNF4*α*) or siRNA control. After 72 h the protein expression of HNF4*α* and PED was measured by western blot (**e**) and *β*-actin served as control. mRNA expression was measured by qPCR (**f**) using RNA 18 S as internal control at 48 h for HuH-7 and 72 h for PLC/PRF/5. Data are reported as mean±S.D. of two independent experiments performed in triplicate. (**g**) SNU-449 cells were transfected with siRNA against HNF4*α* or siRNA against PED alone or in combination, or siRNA control, as indicated. Migration was assessed by CIM plate with xCELLigence apparatus after 12 h and 24 h. Data are reported as mean±S.D. of two independent experiments performed in triplicate. (**h**) Western blot analysis of pERK^Thr202/Tyr204^ and ERK in SNU-449, Hep3B and HuH-7 cell lines transfected with PED-MYC. *β*-Actin was used as loading control. (**i**) pERK^Thr202/Tyr204^ expression in two HCC patients and their non-tumoral counterpart. Calnexin was used as loading control. *P<0.05, **P<0.01, ****P<0.0001

Earlier studies have shown that HNF4*α* supresses PED expression at the mRNA and protein levels by binding to its promoter.^15, 16^ Therefore, we first reconfirmed that HNF4*α* binds to the PED promotor in HCC, as revealed by a luciferase assay in SNU-449 cell lines (Figure 4a). Next, we analyzed HNF4*α* and PED expression in our gene expression microarray of the 59 HCC and matched non-tumoral liver tissues.^17^ We observed a significant inverse correlation between HNF4*α* and PED mRNA expression in the HCCs (Figure 4b). Interestingly, we also observed an inverse correlation between HNF4*α* and PED mRNA expression in the non-tumoral liver tissues of the HCC patients, suggesting that PED regulation by HNF4α is not restricted to liver cancer cells (Figure 4c). In accordance, western blots of PED and HNF4*α* in tumoral and non-tumoral liver tissues of HCC patients also showed an inverse correlation between these two proteins (Figure 4d). Similarly, analysis of a publicly available transcriptome array of transgenic mice (GEO GSE34581)^21^ revealed that hepatic PED expression increased after specifically depleting HNF4*α* in the liver (Supplementary Figure 4A). Moreover, there was an inverse correlation between hepatic PED and HNF4*α* expression (Supplementary Figure 4B). We did not observe a significant difference in HNF4*α* mRNA expression between tumoral and matched non-tumoral tissue in our transcriptome microarray data set (Supplementary Figure 4C). Yet, as described before, HNF4*α* expression significantly decreased in non-tumoral, mostly cirrhotic liver tissue, in comparison to healthy liver samples (*n*=5) (Supplementary Figure 4C), supporting its role in hepatocarcinogenesis.^21, 22^

To investigate if HNF4*α* directly regulates PED expression, we reduced HNF4*α* expression by siRNA in two different liver cancer cell lines (HuH-7 and PLC/PRF-5). After reducing HNF4*α*, protein (Figure 4e) and mRNA levels (Figure 4f) of PED increased in both cell lines. Next, we wanted to test if HNF4*α* regulates cell migration^23, 24^ through PED. Therefore, we performed a rescue experiment and silenced PED and HNF4*α* simultaneously in SNU-449 cells (Figure 4g). As expected, silencing of HNF4*α* alone increased, whereas silencing of PED alone reduced cell migration. A combination of PED and HNF4*α* silencing reverted the suppressive effect of siRNA against PED and cell migration was similar to control transfected cells. Therefore, our experiments indicate that HNF4*α* regulates cell migration through PED in liver cancer cells (Figure 4g).

In addition, we wanted to analyze cellular processes downstream of PED. Earlier studies have revealed that activation of PED leads to an increase of ERK phosphorylation.^25, 26, 27, 28^ Therefore, we increased PED expression by PED-MYC transfection in three different cell lines (SNU-449, Hep3B, HuH-7) and measured total ERK and pERK^Thr202/Tyr204^ expression by western blot. Whereas total ERK expression remained similar, there was a clear increase of pERK^Thr202/Tyr204^ after upregulation of PED (Figure 4h). Detection of pERK^Thr202/Tyr204^ in human HCC tissue samples was technically difficult, but one out of 2 samples already analyzed for PED expression in Figure 4d showed an increase of pERK^Thr202/Tyr204^ in the tumoral tissue (Figure 4i). In conclusion, our results confirm that pERK is one of the downstream proteins activated by PED.

### PED confers resistance to sorafenib

**Figure 5:**
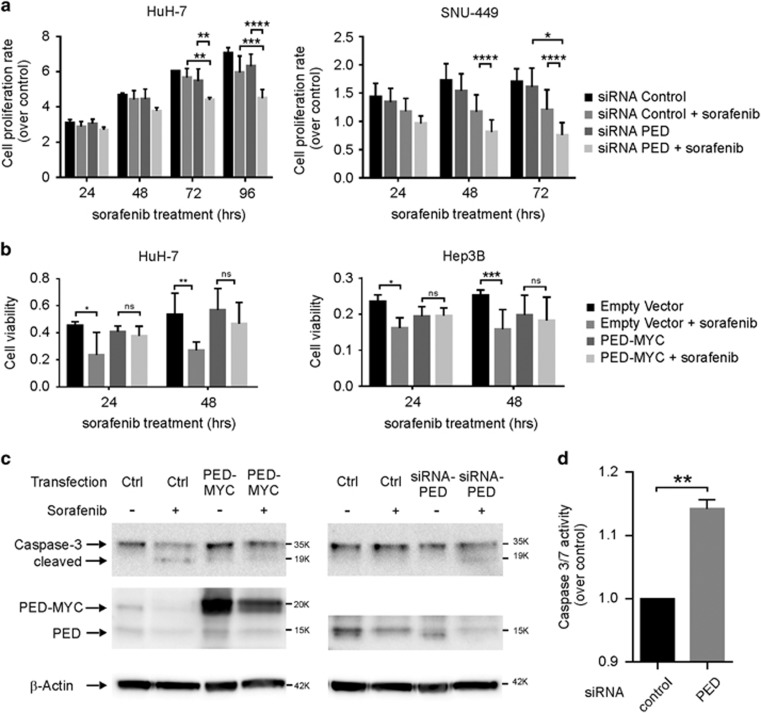
PED confers resistance to sorafenib therapy. (**a**) HuH-7 and SNU-449 cells were transfected with siRNA against PED or siRNA control. Afterwards, HuH-7 and SNU-449 cells were treated with 10 *μ*M and 20 *μ*m respectively of sorafenib or left untreated. Cell growth was evaluated by using the xCELLigence instrument at the indicated time. Data are reported as mean±S.D. of two independent experiments performed in triplicate. (**b**) HuH-7 and Hep3B cells were transfected with PED-MYC vector for 24 h and then seeded in a 96-well plate. 10 *μ*m of sorafenib was added and 24 h or 48 h later, cell viability was measured by a MTT assay. Data are reported as mean±S.D. of two independent experiments perfomed in triplicate. (**c**) HuH-7 cells were transfected with PED-MYC or empty vector (Ctrl) and siRNA against PED or siRNA control (Ctrl), as indicated. After 24 h, cells were treated with 10 *μ*M or 7 *μ*M sorafenib, respectively, for 48 h and caspase-3 activation was measured by western blot. (**d**) HuH-7 cells were transfected with siRNA against PED or control siRNA. Afterwards, cells were treated with 10 *μ*M sorafenib and 48 h later caspase-3/7 assay activation was measured. Data are reported as mean±SD of one experiment performed in triplicate. *P<0.1, **P<0.01, ***P<0.001, ****P<0.0001

Earlier studies in non-HCC cancer cell lines such as breast cancer^29^ and colon cancer^26^ have shown that PED confers resistance to chemotherapy. Therefore, we tested the role of PED in HCC cell lines treated with the multi-kinase inhibitor sorafenib. Sorafenib treatment slightly decreased the proliferation rate of HuH-7 and SNU-449 cells *in vitro* (Figure 5a). However, the effect of sorafenib treatment on cell proliferation became significantly more pronounced after silencing PED expression by siRNA (Figure 5a). *Vice versa*, upregulation of PED in HuH-7 and Hep3B cells by transfection with a PED-MYC vector antagonized the effect of sorafenib on cell viability, whereas sorafenib clearly reduced cell viability in empty vector transfected cells (Figure 5b). Therefore, PED counteracts the effect of sorafenib in HCC cell lines. Western blot and a caspase assay further indicated that the executor caspase-3 (Figure 5c) and caspases 3/7 respectively (Figure 5d) were upregulated after reduction of PED and downregulated after increase of PED in sorafenib treated HuH-7 cells. Therefore, inhibition of apoptosis may be one of the mechanisms by which PED confers resistance to sorafenib treatment

Finally, we exposed ten different HCC cell lines to sorafenib and correlated response rate to PED expression quantified by western blot (Figure 3a; Supplementary Figure 3B; Supplementary Figure 5A). Some cell lines, which were highly sensitive to sorafenib (e.g., HuH-7 and Hep3B) had low PED expression, and other cell lines, which were highly resistant to sorafenib (e.g., SNU-182, PLC/PRF-5 and SNU-449) had high PED expression. However, we did not observe a significant correlation between PED protein expression and sorafenib sensitivity (Supplementary Figure 5B). Therefore, our results confirm that, besides PED, other sorafenib resistance mechanisms exist in HCC cell lines.^30^

## Discussion

The multifunctional phosphoprotein PED has an important role in several cancer entities, yet its expression and function in HCC has not been investigated yet. Our study revealed that PED is overexpressed in HCC at mRNA and protein level. In addition, HCC samples with high PED expression showed an enrichment of a gene signature with poor prognosis and was further associated with shorter survival. Similarly, PED has been reported to be overexpressed in other cancer types such as breast cancer,^29^ lung cancer^31^ and esophageal carcinoma,^32^ where it promotes tumor growth^33, 34, 35^ and is associated with poor survival.^32^ By contrast, it was associated with good prognosis in ovarian cancer when overexpressed.^25^ This difference is mainly explained by its phosphorylation status. PED was unphosphorylated in ovarian cancer.^36^ In contrast, PED was phosphorylated at both serine sites (pSer116, pSer104) in our study. This phosphorylation status indicates an increased ERK1/2 activity and an anti-apoptotic role through FADD.^12^ Therefore, as described before, the phosphorylation status determines if PED acts as a tumor promotor or a tumor suppressor.^13^

Our functional *in vitro* experiments revealed that cell proliferation remained unaffected by PED in liver cancer cell lines. By contrast, cell migration was increased after upregulation of PED and, *vice versa*, decreased after PED reduction. In line with this observation, we noted that HCC samples from patients with metastasis showed higher PED expression. Moreover, PED^high^ tumors showed an enrichement of a gene signature associated with HCC metastasis.^18^ Therefore, our results suggest that PED may promote metastasis formation in HCC by increasing cell migration. Furthermore, PED could be a potential target to prevent metastasis formation, which is associated with very poor prognosis.^37^ Several earlier studies have already shown that PED exerts its effect on migration and invasion by ERK1/2 regulation.^26, 38, 39^ If PED is phosphorylated, as in our study, ERK1/2 is activated with ensuing increase in pERK, which promotes invasion and migration.^38^ By contrast, if PED is unphosphorylated, ERK is sequestered and migration and invasion is reduced, as has been shown for example in colon cancer and neuroblastoma.^26, 40^ We further confirmed that HNF4*α* is an upstream regulator of PED in HCC and binds to the PED promoter. *In vitro* silencing of HNF4*α* increased PED expression with ensuing promotion of cellular migration. In accordance, we detected an inverse correlation between HNF4*α* and PED expression in HCC samples. As a transcription factor, HNF4*α* controls hepatic differentiation, but it also inhibits hepatic proliferation and controls epithelial-to-mesenchymal transition in liver tumors.^41, 42, 43, 44^ Not unexpectedly, HNF4*α* has been shown to have an important role in hepatocarcinogenesis. Upon treatment with diethyl nitrosamine, mice lacking HNF4*α* have an increased liver tumor development. In contrast, rats overexpressing HNF4*α* have a reduced liver tumor development.^22, 41^ By inhibition of the transcription of epithelial-to-mesenchymal transition-regulatory genes such as Snail and Slug, HNF4*α* prevents migration and invasion in HCC.^43, 44^ Therefore, we propose a novel link between HNF4*α* and PED expression in HCC. The downregulation of HNF4*α* during hepatocarcinogenesis leads to an increase of total PED, which becomes phosphorylated. Consequently, ERK1/2 is activated and promotes tumor development and in particular cellular migration.

PED has been shown to mediate chemo resistance in various cancer types such as for example colon cancer and breast cancer.^26, 29^ In HCC, sorafenib is currently the only drug approved for systemic treatment.^45^ However, it goes frequently along with adverse side effects and resistance.^8^ Furthermore, it has limited treatment efficacy. Interestingly, silencing of PED sensitized HuH-7 and SNU-449 cells to sorafenib treatment, whereas upregulation of PED counteracted sorafenib effect in Hep3B and HuH-7 cells. In detail analysis suggest that PED modulates apoptotic caspase cascade and indicates that the observed PED overexpression in HCC may prevent the apoptotic effects of sorafenib treatment. In line with our observations on the functional role of PED, earlier studies have revealed that epithelial–mesenchymal transition as well as ERK1/2 are involved in sorafenib resistance.^8^ In conclusion, measuring PED expression could represent a marker to predict sorafenib treatment response.

In summary, our study shows that high PED expression in HCC is associated with poor survival and promotes migration of cancer cells. Furthermore, PED expression reduces the effect of sorafenib, which opens new perspectives in understanding sorafenib resistance in HCC patients. Furthermore, it suggests that co-targeting of PED may improve the efficacy of sorafenib.

## Materials and methods

### Patients

All tissue specimens were collected from the archive at the Institute of Pathology, University Hospital of Basel, Switzerland. The collection protocol conforms to ethical guidelines of the 1975 Declaration of Helsinki and has been approved by the ethics committee of the Kanton Basel (Ethikkommission beider Basel). Written informed consent was obtained from all participants.

### Tissue microarray

For TMA construction, a representative tumor area was selected on an hematoxylin and eosin (H&E)-stained slide of the donor block. A core punch with a diameter of 0.6 mm was taken from the tumor (*n*=45) and in selected cases from the non-tumoral liver tissue (*n*=20) of each slide. Core punches were transferred to a new paraffin recipient block using a programmed tissue arrayer (Beecher Instruments, Silver Spring, MD, USA).

### Immunohistochemistry

For immunohistochemistry, 4 *μ*m slides obtained form the TMA were stained with a polyclonal sheep PED antibody (AF5588, R&D System, Minneapolis, USA) using the Dako Real Detection System (Agilent Technologies, Santa Clara, CA, USA). In brief, sections were first blocked with Dako Envison FLEX/Peroxidase-Blocking Reagent for 5 min and stained thereafter with primary anti-PED antibody (1:50) for 30 min. After washing, biotinylated secondary antibody was added (anti-sheep IgG, Vector Laboratories, Burlingame, CA, USA; BA-6000, dilution 1:1000) and detected using streptavidin-horseradish peroxidase conjugate (Agilent Technologies) and DAB+ Chromogen (Agilent Technologies). PED cytoplasmic staining intensity was evaluated by a board-certified pathologists with expertise in hepatopathology (MSM) and graded semi-quantitatively into: 0 for negative staining, 1+ for weak positive staining, 2+ for moderate positive staining and 3+ for strong positive staining, as shown representatively in Supplementary Figure 1. The *h*-score was calculated by multiplying staining intensity with percentage of positive tumor cells.

### RNA extraction and quantitative real-time PCR

Total RNAs from fresh frozen tissues were extracted using Trizol (LuBioScience GMbH, Lucerne, CH, USA) according to the manufacturer’s protocol. 500 ng of total RNA were reverse transcribed using first cDNA synthesis beads (GE Healthcare, Little Chalfont, UK). mRNa expression of *PED* and *18 SRNA* (used as an internal reference) was assessed using the TaqMan® Probe-Based Gene Expression Analysis and the (Assay-on-Demand: Hs00269428_m1 and Hs99999901_s1, respectively; Life Technologies, ThermoFisher Scientific) according to the manufacturer’s protocol.

*HNF4α* mRNA quantification was performed with SybrGreen Master Mix (ThermoFisher Scientific) using the following primers covering the gene of interest and the reference control (*18 S*): *HNF4α* FWD 5′–3′: TCAACCCGAGAAAACAAA; *HNF4α* REV 5′–3′: ACCTGCTCTACCAGCCAGAA; *18 S*-FWD: 5′–3′: AACCCGTTGAACCCCATT; *18S-*REV 5′–3′: CCATCCAATCGGTAGTAGCG. For relative quantization the 2^(−ΔCT)^ or the 2^(−ΔΔCT)^ method was employed as previously described.^46^ All reverse-transcriptase reactions, including no-template controls, were run in triplicate on an Applied Biosystem Viia VII real-time PCR system and target gene expression levels were normalized to thr reference gene. Data analysis was performed using the build-in Applied Biosystem dedicated software (Life Technologies, ThermoFisher Scientific).

### Protein isolation and western blotting

Protein extraction and western blotting were performed as previously described.^47^ Primary antibodies used were: anti-*β*-Actin (Sigma-Aldrich, St. Louis, MO, USA), anti-PED,^48^ anti-Calnexin (CellSignalling Technology, Basel), anti-PED S116 (ThermoFisher Scientific) anti-PED S104 (Cell Signaling Technologies, Danver, MA USA), anti-Caspase-3 (Cell Signaling Technologies, Danver, MA USA), anti-total ERK and anti-phopsho ERK (Cell Signaling Technologies), and anti-HNF4*α* (Santacruz Biotechnologies, Heidelberg, Germany for cell lines and Novus Biological, Oxon, England, UK for human samples). Blots were visualized by using the Azure c3000 (Azure Biosystems, Inc., Dublin, CA, USA).

For human tissue samples the following protocol was used. Frozen tissue was crushed into a fine powder in metal mortar (constructed in-house) cooled on dry ice and resuspended in 8 M Urea (Applichem, A1086) containing 50 mM Tris-HCl, 150 mM NaCl, 1 mM PMSF, 1X Complete Mini Protease Inhibitors from Roche (Roche, Basel, Switzerland), 1X PhosSTOP (Roche) and homogenized using a Polytron (PT 10-35 GT, Kinematica, Lucerne, Switzerland) at 500 rpm for 2 min on ice. The lysates were then subjected to rotation at 4 °C with intermittent vigorous vortexing and then were centrifuged at 10 000 × *g* for 10 min at 4 °C to remove cell debris. The protein concentration in the supernatants was determined by the Bradford assay using bovine albumin as reference. Human tissue samples were all from resection specimens of patients operated for HCC. Mean age of patients was 66.8 years, mean tumor diameter was 9.6 cm.

Protein levels were quantitatively assessed by a densitometric analysis using ImageJ analysis software (Rasband, W.S., ImageJ, U. S. National Institutes of Health, Bethesda, MD, USA, http://imagej.nih.gov/ij/, 1997–2016).

### Cell lines and transfection

All cells were purchased from the American Type Culture Collection (ATCC) and maintained in 5% CO_2_-humidified atmosphere at 37 °C. Media were supplemented with 10% heat-inactivated fetal bovine serum (FBS) 10%, 2 mM Glutamine and 100 U/ml penicillin/streptomycin. Transfections were performed using Lipofectamine 3000 (Life Technologies, ThermoFisher Scientific, Reinach, Switzerland) according to manufacturer’s protocol. For PED overexpression, the pcDNA-3-PED-MYC^48^ plasmid was used. PED silencing was achieved with On-Target Smart Pool for PEA15 (Ge Dharmacon, Freiburg im Breisgau, Germany) and the relative negative control was used. For HNF4*α* silencing, a specific siRNA and its relative control were used (SantaCruz Biotechnologies, Heidelberg, Germany). All cell lines were confirmed negative for mycoplasma infection using the PCR-based Universal Mycoplasma Detection kit (American Type Culture Collection, Manassas, VA, USA) as previously described.^47^

### Proliferation assay and sorafenib sensitivity

Cell Proliferation was assessed using the xCELLigence system (OLS, Basel, Switzerland). 5000 cells per well were plated and then transfected 24 h after. For sorafenib treatment, 10 or 20 *μ*M as indicated of sorafenib was added to the cells 24 h after transfection. For screening sorafenib sensitivity in HCC cell lines, including in HuH-7 and Hep3B cells upon PED overexpression, the 3-(4, 5-dimethylthiazol-2-yl)-2, 5-diphenyltetrazolium bromide (MTT) assay was adopted.

### Luciferase reporter assay

pGL3 basic vector, PED-477 luciferase promoter and TK-renilla luciferase plasmid hve been described before.^16^ SNU-449 cells were transfected in 96-well plate with 20 ng of Renilla luciferase and 100 ng of Firefly Luciferase constructs in combination or not with the siRNA for HNF4*α* using lipofectamine 3000 following the manufacturers protocol. After 48 h luciferase assay was performed using Dual-GLO Luciferase® Assay System (Promega, Madison, WI, USA) on a multiwall plate reader (Synergy H1, Biotek, Luzern, Switzerland). Transfection of each construct was performed in triplicate. Ratios of Renilla luciferase readings to Firefly luciferase readings were taken for each experimental point.

### Caspase-3/7 activity measurement

5x10^3^ cells were seeded in a 96-well plate and then transfected after 24 h. The day after 10 *μ*M of sorafenib was added to the plate fand then caspase-3/7 activity was measured after 48 h with the Apo-ONE® Homogeneous Caspase-3/7 assay (Promega, Dübendorf, Switzerland) according to the manufacture instruction.

### Migration assay

Transwell Permeable Supports, 8 *μ*m pore size (Corning Incorporate, Corning, USA) were used to perform migration assay. 10^5^ cells were resuspended in medium with 1% FBS and seeded in the upper chamber. After 24 h, cells were stained with 0.1% Crystal Violet in 25% methanol and then eluted in 1% SDS.^49^ For rescue experiment, migration was assessed with CIM plates using the xCELLigence system (OLS, Basel, Switzerland). 48 h after transfection, cells were detached and counted. 3x10^4^ cells were plated in each well according to manufacturer’s instruction and migration was assessed 12 and 24 h after seeding.

### PED mRNA expression analysis by microarray and GSEA

Data from the gene expression profiling (GeneChip^®^Human Gene 1.0ST array, Affymetrix) of 59 needle biopsies from HCC tissues and their corresponding non-tumoral liver tissues was obtained from a previous study^17^ and re-analyzed using the Qlucore software (Lund, Sweden). GSEA^50^ was performed to assess the enrichment of previously published metastasis or prognosis-associated gene signatures between PED^high^ and PED^low^ HCCs. A two-fold change or more in PED expression between tumoral and non-tumoral tissues was regarded as cut-off to classify patients into PED^high^ and PED^low^ subclasses.

### Statistical analysis

Chi-square test (*χ*^2^-test), Fisher's exact test and Mann–Whitney *U*-test for nonparametric variables and Student’s *t*-test for parametric variables were used for statistical analyses. Patient survival was assessed using the Kaplan–Meier method and the log-rank test. All tests were two-sided with *P*-values <0.05 were considered statistically significant. Analyses were performed using GraphPad PRISM (GraphPad Software, La Jolla, USA).

## Publisher’s Note

Springer Nature remains neutral with regard to jurisdictional claims in published maps and institutional affiliations.

## Figures and Tables

**Table 1:** Clinico-pathological features of the TMA cohort

**Hepatocellular carcinoma (*n*=45)**	**Frequency (%)**
Age (years), median (range)	69 (10–84)
*Sex*	
Female	9 (20)
Male	36 (80)
*Tumor grade (Edmondson)*	
G1	0 (0)
G2	18 (40)
G3	24 (53)
G4	3 (7)
*pT stage*	
pT1	19 (42)
pT2	12 (27)
pT3	9 (20)
NA	5 (11)
