# Supplementary material for: Phosphoprotein enriched in diabetes (PED/PEA15) promotes migration in hepatocellular carcinoma and confers resistance to sorafenib
Source: Cell Death Dis. 2017 Oct 26;8(10):e3138–. doi: 10.1038/cddis.2017.512 (PMC5682677; doi:10.1038/cddis.2017.512)
Supplement: Supplementary Figure Legends [file cddis2017512x1.docx]

**SUPPLEMENTARY FIGURE LEGENDS**

**Supplementary Figure 1. Staining pattern of PED in different HCC specimens**

Representative examples of nuclear immunohistochemical detection of PED in HCC tissues with **(A)** negative (0), **(B)** weak (1+), **(C)** moderate (2+) and **(D)** strong (3+) staining intensity. Black bar corresponds to 20μm.

**Supplementary Figure 2. PED expression in HCC samples. (A)** PED mRNA expression in HCCs (n=24) and corresponding non-tumoral liver tissues (NT) was measured by qRT-PCR in the same samples analyzed for gene expression array with sufficient RNA left. Data are reported as 2^(-ΔΔCT)^. **(B)** Western blot analysis for total PED expression in three HCC patients. Ponceau staining was used as loading control. ** p<0.001

**Supplementary Figure 3. PED expression in liver cancer cell lines. (A)** Densitometry analysis of PED expression in 10 different HCC cell lines. Data were normalized to β-Actin intensity **(B)** qRT-PCR analysis of PED mRNA expression in various liver cancer cell lines. 18s RNA was used as an internal control. Data are reported as 2-ΔCt ± SD. **(C)** Western blot analysis of PED expression 72 hours after transfection of HuH-7, HLE, SNU-449, PLC/PRF/5 and Hep3B cell lines with PED-MYC plasmid (+) or an empty vector (-). β-Actin was used as loading control. **(D)** Western blot analysis of PED expression 72 hours after transfection of HuH-7 and SNU-449 cell lines with siRNA against PED (+) or a control siRNA (-). β-Actin was used as loading control.

**Supplementary Figure 4.** **PED is inversely correlated to HNF4α** **expression**. **(A)** PED expression in liver-specific tamoxifen-induced HNF4α knock-out mice and respective controls (GEO GSE34581). One sample from the tamoxifen group was excluded because it was considered as an outlier. **(B)** Correlation between PED and HNF4α expression in mice treated with tamoxifen or control. Spearman test indicated an inverse correlation between PED and HNF4α expression (r= -0.5, p=0.012). **(C)** HNF4α expression levels in healthy liver (n=5), HCC samples and their matched non-tumoral (NT) counterpart measured in a transcriptome gene expression microarray as described above. Data are reported as probe intensity.

**Supplementary Figure 5. (A)** 10 different HCC cell lines were treated for 48 hrs with 20μM of sorafenib (grey) or left untreated (black). MTT assay was used to assess cell viability. Data are reported as mean±SD of two independent experiments performed in triplicate. Cell viability of treated cell line in percentage of untreated cell lines is indicated above bars. **(B)** Correlation between PED expression measured by densitometry analysis of western blot and sorafenib sensitivity in 10 different liver cancer cell lines. No direct correlation between PED and sorafenib sensitivity (r= 0.02, p=0.68; spearman test).
