# Supplementary figures and images for: Phosphoprotein enriched in diabetes (PED/PEA15) promotes migration in hepatocellular carcinoma and confers resistance to sorafenib
Source: Cell Death Dis. 2017 Oct 26;8(10):e3138–. doi: 10.1038/cddis.2017.512 (PMC5682677; doi:10.1038/cddis.2017.512)

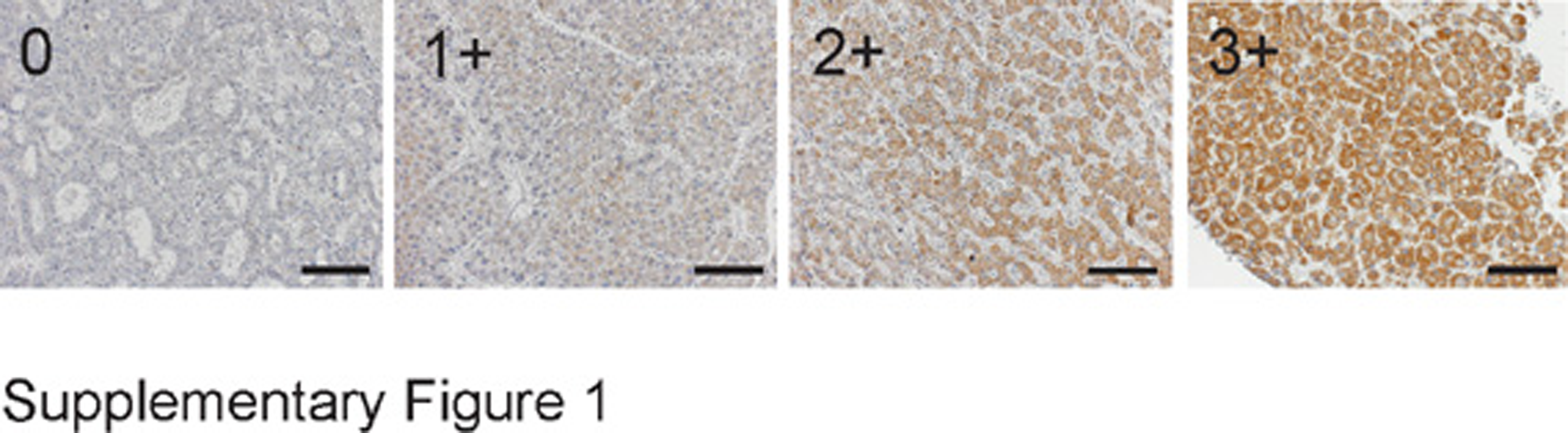

Supplement: Supplementary Figure 1 [file cddis2017512x2.tif]

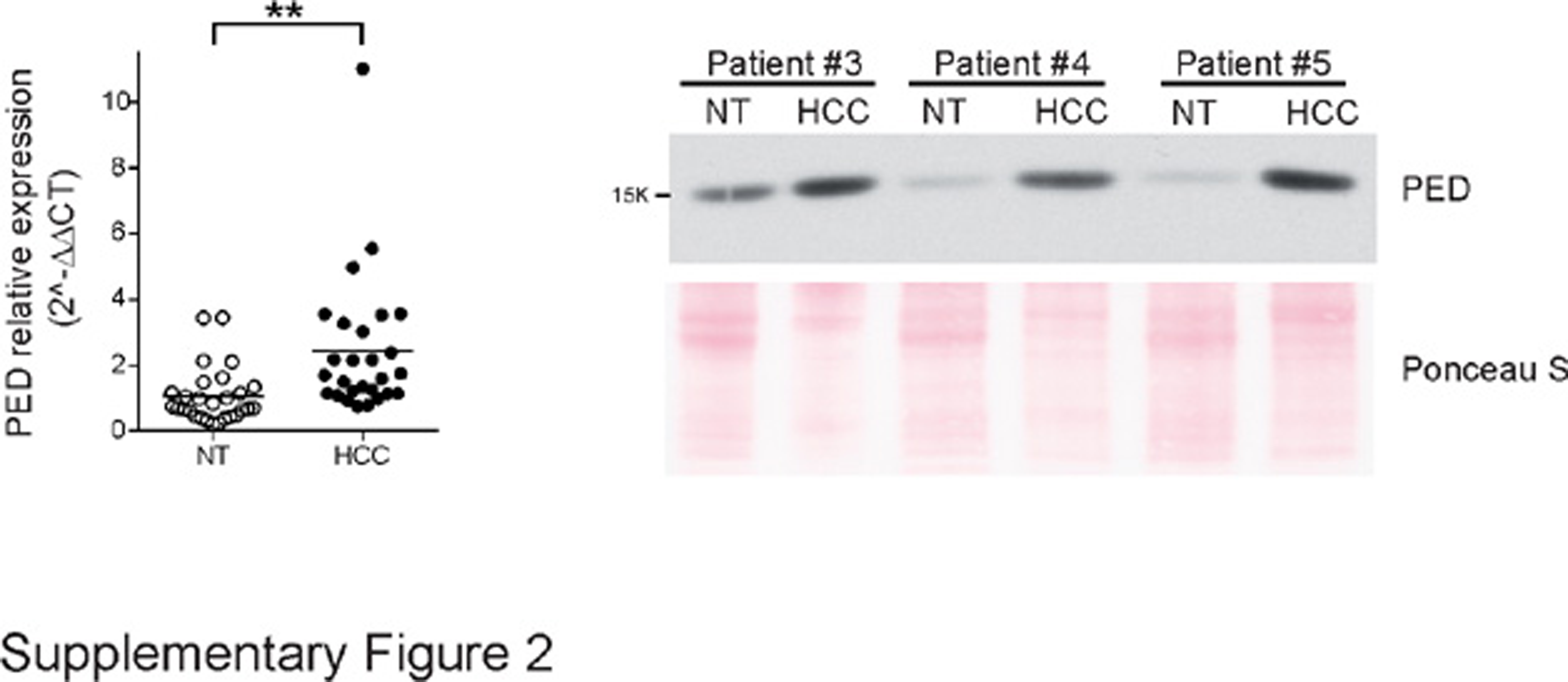

Supplement: Supplementary Figure 2 [file cddis2017512x3.tif]

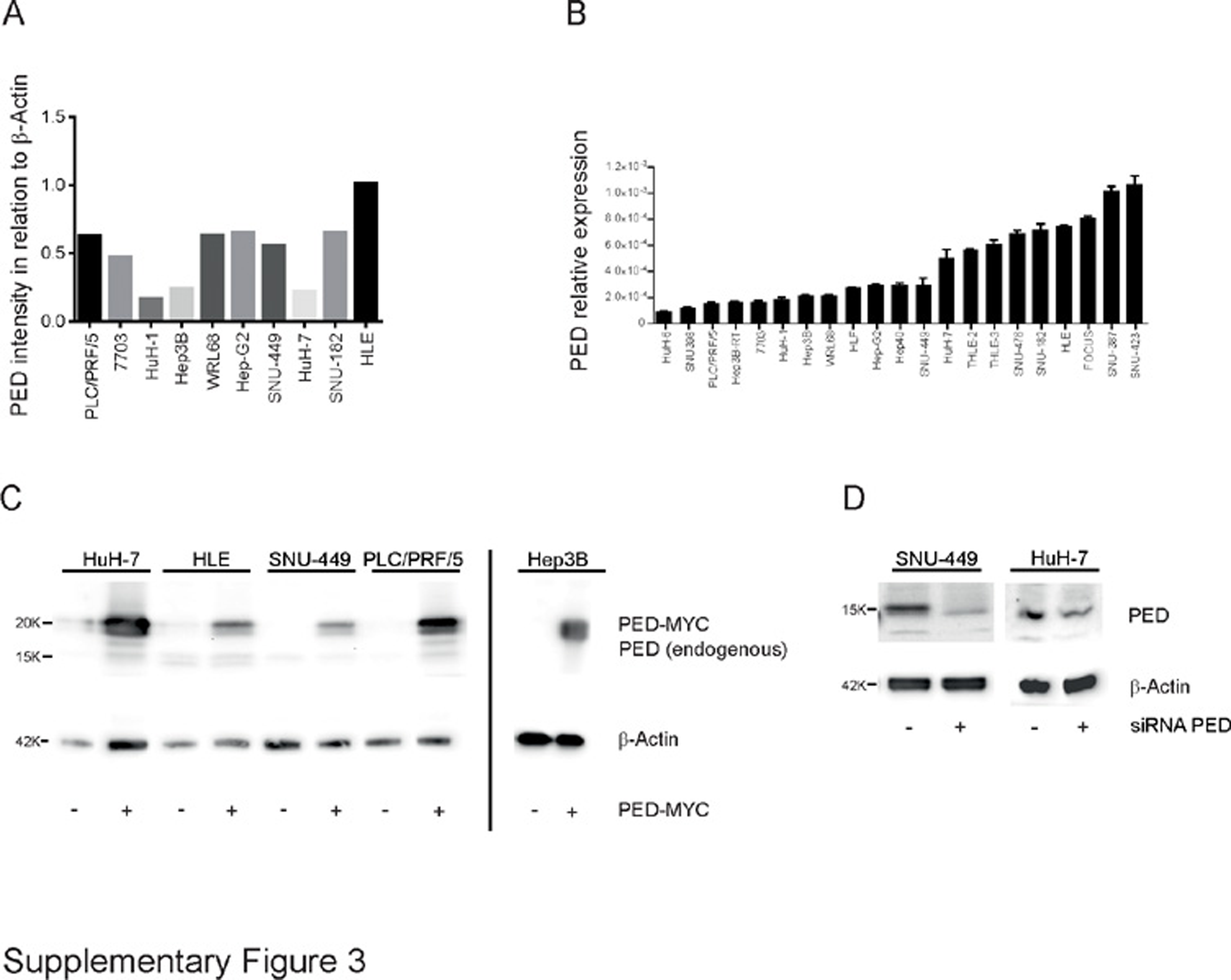

Supplement: Supplementary Figure 3 [file cddis2017512x4.tif]

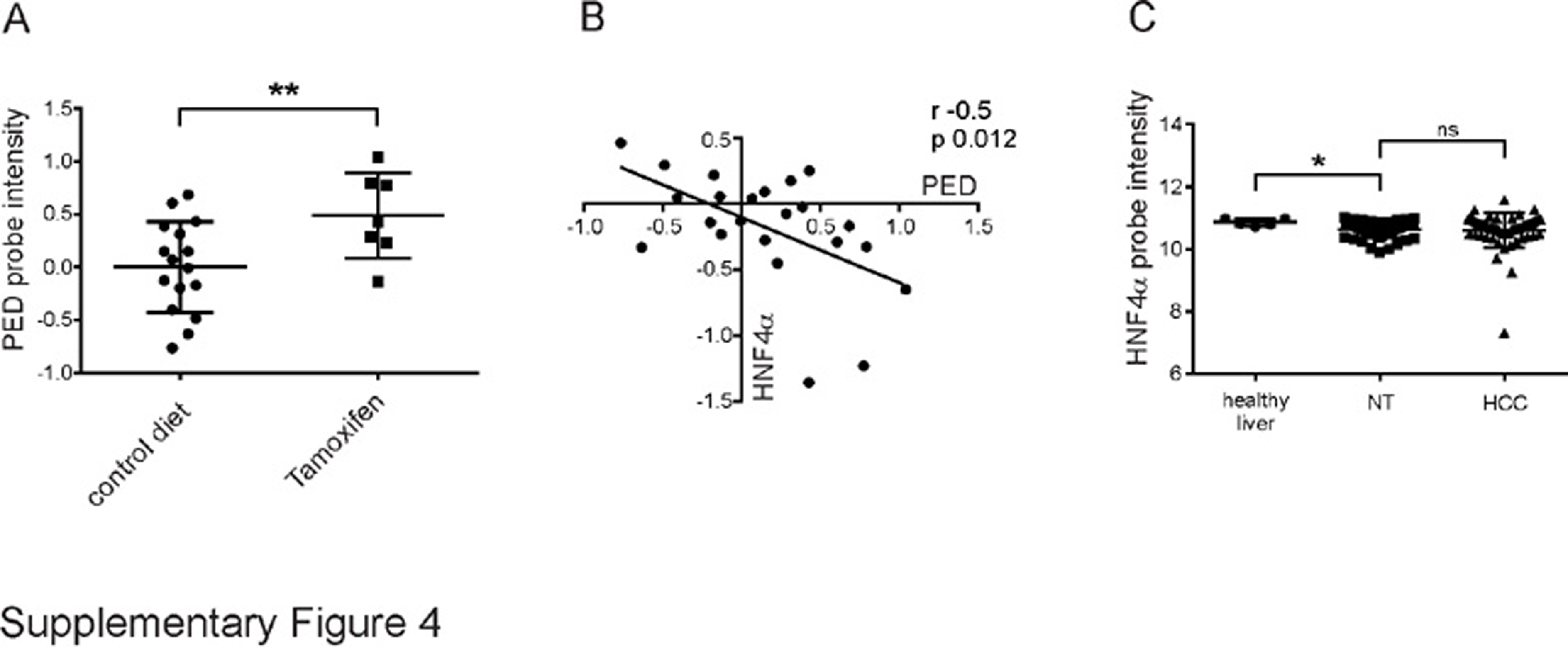

Supplement: Supplementary Figure 4 [file cddis2017512x5.tif]

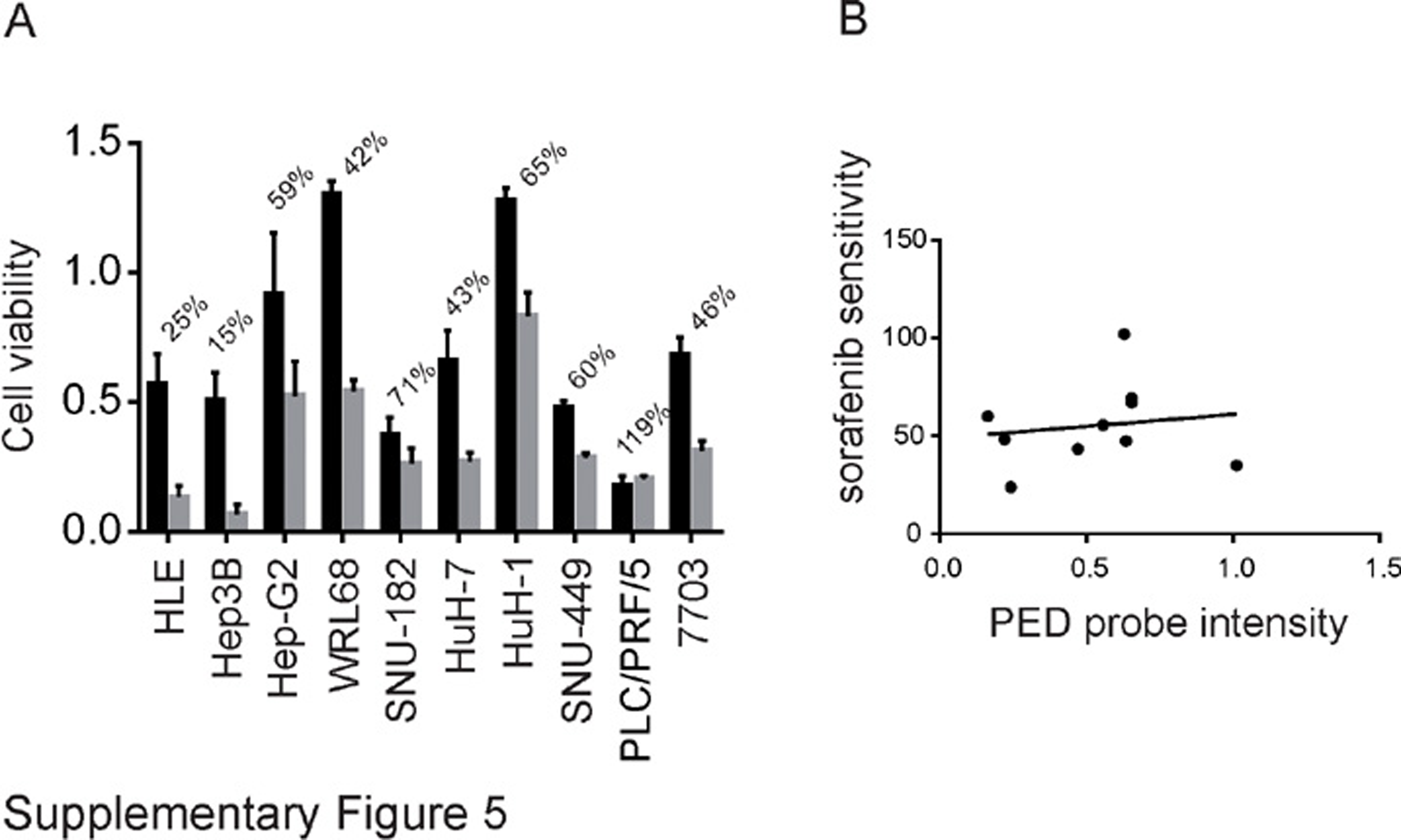

Supplement: Supplementary Figure 5 [file cddis2017512x6.tif]
